# Prevalence and Determinants of Low Birth Weight in India: Findings From a Nationally Representative Cross-Sectional Survey (2019-21)

**DOI:** 10.7759/cureus.36717

**Published:** 2023-03-26

**Authors:** Siaa Girotra, Neha Mohan, Mansi Malik, Shubhanjali Roy, Saurav Basu

**Affiliations:** 1 Indian Institute of Public Health - Delhi, Public Health Foundation of India, New Delhi, IND

**Keywords:** maternal-child health, antenatal care visits, nfhs-5, survey research, maternal health, low birth-weight

## Abstract

Introduction: Low birth weight (LBW) is an important public health indicator extensively linked to infant and child mortality, especially in lower-middle-income countries (LMICs). Globally, 15.5% of all infants are born with LBW while 95% of these occur in LMICs. This study aims to examine the prevalence and determinants of LBW in India.

Methods: Data were obtained from the fifth National Family Health Survey (NFHS) round conducted during 2019-2021. The study sample included women aged 15-49 years who had a singleton pregnancy in the five years preceding the survey (N=175,240). A bivariate analysis was carried out and a logistic regression model was fitted to assess the maternal determinants affecting the birth weight among newborns.

Results: A total of 175,240 mothers were included in the present study. The proportion of newborns with LBW was 17.29% (n=26366, 95% confidence interval [CI] 17.01, 17.57), of which 6% (n=1450, 95% CI 5.61, 6.41) had very low birth weight (less than 1500 g). An increase in the education level of women or wealth index also resulted in significantly reduced odds of LBW in the newborn. However, the number of antenatal care (ANC) visits lacked any statistically significant association with the odds of having a newborn with LBW.

Conclusions: The burden of LBW in India in recent years has remained stable despite impressive economic growth and increased public health spending on food security and nutritional supplementation. Strengthening the quality of ANC services for pregnant women with a focus on sensitization and awareness generation for improving maternal nutrition requires high prioritization.

## Introduction

Low birth weight (LBW) is one of the most important public health indicators of antenatal care (ANC), maternal health and nutrition, and economic vulnerability especially in lower-middle-income countries (LMICs) [1]. It reflects the poor trajectory of intrauterine growth and is considered the most important determinant linked to infant and child mortality, well-being, and survival [2]. A high burden of LBW secondary to adverse social determinants, especially undernutrition, which is widely prevalent in mothers in LMICs, creates a vicious cycle of poverty, ill health, and high treatment costs [3].

The World Health Organization has defined LBW as “birth weight of less than 2500 g” irrespective of gestational age with measurement of the weight of the newborn taken within 1 hour of birth to eliminate any significant postnatal weight loss [4]. LBW is further categorized into very low birth weight (weight < 1500 g) and extremely low birth weight (weight < 1000 g) [5]. LBW is a major determinant of perinatal survival and early neonatal morbidity and mortality [6,7].

The global prevalence of LBW is 14.6% (12.4-17.1) accounting for 25 million LBW infants born every year, of which 95% occur in LMICs with South Asia accounting for nearly 52% of the global burden [8]. The prevalence of LBW in India estimated from nationally representative survey data reduced from 22% (National Family Health Survey [NFHS-3], 2005-6) to 17.5% (NFHS-4, 2015-16) [9] but the country is still not on track toward achieving the targeted 30% reduction in LBW burden by 2025 [10]. 

LBW is a multifactorial phenomenon with several known maternal and fetal risk factors [11]. Maternal factors associated with LBW are maternal age (<16 years and >40 years), multiple pregnancies, obstetric complications, trauma, pre-eclampsia or eclampsia, certain infections, chronic maternal conditions (hypertension, diabetes), nutritional status, and substance abuse (smoking, alcohol) [1,11-13]. The fetal factors that can be linked with LBW are intrauterine growth retardation, fetal infection and anomalies, and some placental conditions [14]. LBW newborns are at a high risk of developing hypothermia and hypoglycemia and are at a higher risk of early death apart from having a lifelong susceptibility to developing long-term neurological and language impairments [15]. Additionally, LBW accentuates the risk of early onset and incidence of chronic diseases including cardiovascular disorders, diabetes, and dyslipidemia in adult life [16]. 

The government of India and state governments have focused on strengthening national health programs to address the challenge of LBW newborns in Indian mothers. These programmatic initiatives in recent years include enhanced focus on improving the quality of ANC services and reduced out-of-pocket costs (Janani Shishu Suraksha Karyakram; Reproductive, Maternal, Newborn, Child, and Adolescent Health program), and supplementary nutrition services for pregnant mothers with weight gain monitoring (Integrated Child Development Services Scheme and POSHAN Abhiyan) [17]. The public health impact of these current initiatives in India can be understood by estimating the change in the prevalence of LBW through updated survey data. The NFHS (fifth round, 2019-21) is a large nationally representative cross-sectional survey that provides national-, state-, and district-level estimates of demographic, reproductive, newborn, and child health indicators [18].

Therefore, this secondary data analysis of NFHS-5 was conducted with the objective of determining the prevalence of LBW babies and their determinants in India. The findings from this assessment can be used to design evidence-based interventions that can be incorporated into the ongoing government programs to lower the burden of this major public health challenge.

## Materials and methods

Data source

The data were obtained from the NFHS-5 conducted during 2019-21. A stratified, two-stage sample design is adopted in the NFHS-5 with primary sampling units being census enumeration blocks (CEBs) in urban areas and villages in rural areas. Villages were chosen from the sampling frame inside each rural stratum with a probability proportional to size (PPS) whereas the Office of the Registrar General and Census Commissioner, New Delhi, provided CEB data for urban regions. The sample CEBs were chosen using PPS systematic sampling. The fifth round of NFHS gathered information from 636,699 households, including 724,115 women and 101,839 men. Face-to-face interviews were conducted with all the participants by trained enumerators using a standardized interview schedule [18]. Data from women aged 15-49 years who had a singleton pregnancy in the last five years were included in this analysis because newborns in multiple births had a disproportionately greater likelihood of LBW. In the NFHS-5 sample, among the mothers who underwent childbirth in the past five years, less than 1% (n=1637) had multiple births, and within these newborns, 64% (n=887) were having LBW. 

Outcome variable

The primary outcome variable was the birth weight of the newborn. Information on birth weight in NFHS-5 was obtained from the written record in 55.96% of cases, from the mother's recall in 35.42% of cases, while it was unknown in 8.62% of cases [18]. The birth weight variable was further dichotomized as "low birth weight" if it was less than 2.5 kg and "normal birth weight" if equal to or more than 2.5 kg (as per WHO definition) [10].

Independent variables

Well-established factors known to affect newborn birth in the existing literature were examined for their association with the outcome variable. Individual-level factors such as age, marital status, education level of mother and father, occupation, and other lifestyle factors such as mass media exposure and tobacco or alcohol consumption were included. Household characteristics like religion, caste, health insurance type, wealth index, and household size were also considered along with pregnancy characteristics such as parity and the intention of pregnancy.

Certain composite independent variables were developed:

a. Mass media exposure: Categorized as "Yes" for mothers who either read newspapers or magazines or listened to the radio or watched television compared to those who did not.

b. Dietary diversity index (DDI) in mothers: Categorized as "Low," "Medium," and "High" by measuring the eating frequency of nine food items: milk or curd, pulses or bean, dark green leafy vegetables, fruits, eggs, fish, chicken or meat, fried food, and aerated drinks. Responses were recorded as daily, weekly, occasionally, and never for each food item. These were dichotomized as consumers (daily, weekly, and occasionally) and non-consumers (never). These scores were added to get the final DDI - low (consumed <3 food items), medium (consumed 3-6 food items), and high (consumed 7-9 food items).

c. Health insurance: Categorized as government, private, or none. Employees’ State Insurance Scheme (ESIC), Central Government Health Scheme (CGHS), any state health insurance schemes, Rastriya Swasthya Bima Yojana (RSBY), and community health insurance programs were combined as government insurance while other health insurance through employer medical reimbursement from employer and other privately purchased commercial health insurance were collapsed as private insurances.

The independent variables with several categories were collapsed into meaningful alternatives. Marital status was integrated into three categories - unmarried, married, divorced/separated/widowed. The occupation of the mothers was categorized as working or non-working. The place of delivery was categorized as public, private, and at home. The community was classified as Scheduled Caste (SC), Scheduled Tribe (ST), Other Backward Castes (OBCs), and others. The SC and ST communities are officially considered as representing historically the most socioeconomically disadvantaged groups in India.

The region variable was classified based on the state variable as follows:

North: Jammu & Kashmir, Himachal Pradesh, Punjab, Chandigarh, Uttarakhand, Haryana, NCT of Delhi, Rajasthan, and Ladakh.

Central: Uttar Pradesh, Madhya Pradesh, and Chhattisgarh.

East: Bihar, West Bengal, Jharkhand, and Orissa.

Northeast: Sikkim, Arunachal Pradesh, Nagaland, Manipur, Mizoram, Tripura, Meghalaya, and Assam.

West: Gujarat, Dadra & Nagar Haveli, Daman & Diu, Maharashtra, and Goa.

South: Andhra Pradesh, Karnataka, Lakshadweep, Kerala, Tamil Nadu, Puducherry, Andaman & Nicobar Islands, Telangana.

Statistical analysis

The biological plausibility of all data points was checked before proceeding with the analysis. The sample included all women aged 15-49 years who had a singleton pregnancy in the five years preceding the survey. The sample thus consisted of 175,240 women. Independent variables were first described descriptively after setting up the data for survey analysis. All the weighted percentages (using the “svy” suffix) along with the frequencies were reported for each exposure variable.

Observations were made to "missing" wherever "don’t know" was present as a category. We performed logistic regression to calculate the odds ratio at a 5% level of significance. Variables with chi-square p-value <0.05 of the crude model were carried forward in the adjusted model. Adjusted logistic regression was performed to evaluate the independent effect of each factor variable on the outcome. The model assumption of linear association between the explanatory variables and log odds of outcome was checked and there was no issue of multicollinearity with the dataset. Data analysis was performed in STATA version 15.1 (Stata Corporation, College Station, TX, USA).

Ethics statement

This study is the secondary data analysis of publicly available NFHS-5 data. The survey's participants voluntarily and knowingly gave their written and informed consent. The International Institute of Population Sciences (IIPS) ethical review board granted the survey its ethical approval. After reviewing the submitted proposal, DHS (Demographic Health Survey) granted access to the dataset to the investigators with permission to conduct this analysis. 

## Results

A total of 175,240 mothers were included in the present study. The weighted prevalence of newborns with LBW was 17.29% (n=26366, 95% CI 17.01, 17.57), of which 6% (n=1450, 95% CI 5.61, 6.41) had very low birth weight (VLBW, defined as birth weight less than 1500 g). Overall, the proportion of newborns with VLBW was 2.28% (95% CI 2.18, 2.38). 

In the sample population, a large proportion (71.39%) of mothers belonged to the 21-30 age group and the majority (91%) were married. Nearly half (51%) of the women were educated up to secondary (middle) school. Nearly two in three (66%) women reported moderate diversity in their diet. Less than 1% reported consuming alcohol and 3% reported tobacco smoking. Nearly 22% of the women belonged to the poorest wealth quintile (Table 1).

**Table 1:** Sociodemographic characteristics of women who experienced pregnancy in the past five years in India

Characteristic	n	%
Age		
15-20	11,125	6.95
21-30	122,112	71.39
31-49	42,003	21.67
Marital status		
Unmarried	275	0.10
Married	172,358	98.75
Widowed/Separated/Divorced	2607	1.15
Highest educational level		
No education	35,667	19.54
Primary	21,545	11.74
Secondary	91,829	51.50
Higher	26,199	17.22
Husband’s education (n=26,597)		
No education	3813	13.84
Primary	3347	12.78
Secondary	14,943	55.30
Higher	4494	18.08
Occupation (n= 26,717)		
Working	6799	21.98
Not working	19,918	78.02
Lifestyle factors		
Mass media exposure frequency		
No exposure	48,594	26.79
Any exposure	126,646	73.21
Dietary diversity index		
Low	22,482	11.71
Moderate	116,713	66.28
High	36,045	22.02
Drinks alcohol		
Yes	2775	0.55
No	172,465	99.45
Tobacco consumption (smoking/smokeless)		
Yes	10,991	3.25
No	164,249	96.75
Household characteristics		
Religion		
Hindu	128,747	79.57
Muslim	25,010	15.92
Other	21,483	4.51
Caste (n=165,351)		
Scheduled Caste	34,975	24.08
Scheduled Tribe	35,097	10.49
Other Backward Castes	66,388	45.63
Others	28,891	19.81
Health insurance		
Government schemes	31,978	16.51
Private funded	16,113	7.30
None	127,149	76.19
Type of residence		
Urban	37,574	28.16
Rural	137,666	71.84
Region		
North	33,226	13.60
South	22,513	16.95
Central	43,366	26.66
East	33,075	25.82
West	15,789	12.91
Northeast	27,271	4.06
Wealth index		
Poorest	44,488	22.80
Poor	40,146	21.06
Middle	34,274	19.59
Richer	30,758	19.23
Richest	25,574	17.32
Household size		
≤5	85,803	48.26
≥6	89,437	51.74

The clinical characteristics of the participants are reported in Table 2. A majority (53%) of the women reported availing of ≤4 ANC visits during their previous pregnancy and nearly two in three (65%) women had ≥2 children.

**Table 2:** Clinical characteristics of women who experienced pregnancy in the past five years in India

Characteristic	n	%
Pregnancy intended (n=175,206)		
Yes	162,044	92
No	13,162	8
Parity		
1	59,626	34.60
>2	115,614	65.40
Anemia (n=168,745)		
Severe	3981	2.20
Moderate	50,930	30.53
Mild	43,911	26.46
Non-anemic	69,923	40.81
Previous miscarriage/abortion/stillbirth (n=175,240)		
Yes	17,544	10.83
No	157,696	89.17
Place of delivery (n=175,206)		
Public hospital	114,093	63.03
Private hospital	39,649	28
At home	21,077	9.76
Other	387	0.22
Type of delivery		
Caesarean section	37,085	23.77
Non-caesarean section	138,121	76.23
Sex of child		
Male	94,123	53.93
Female	81,117	46.07
Number of antenatal care visits (n=161,497)		
≤4	88,333	53.15
≥5	73,164	46.85

On bivariate analysis, the following variables had significantly lower odds of LBW with a statistically significant association: maternal age 20-30 years, women with higher educational levels, belonging to a non-SC community, belonging to non-Hindu religion, belonging to higher wealth quintiles, exposure to mass media, moderate or higher dietary diversity, tobacco smoking, living in rural areas, previous history of miscarriage, less than four ANC visits, having any health insurance, absence of severe anemia, higher parity (≥2), delivery in a public facility, and gender being a male child. 

The above variables were included in a logistic regression model. However, on adjusted analysis, the number of ANC visits and maternal age were not significantly associated with the occurrence of LBW in the newborn. Compared to women who delivered at a public hospital, the odds of having an LBW newborn were higher in women who delivered at a private facility (adjusted odds ratio [aOR]=1.12; 95% CI 1.06, 1.18; p<0.001) or at home (aOR=1.20; 95% CI 1.11, 1.30; p<0.001). Compared to male newborns, female newborns had significantly higher odds (aOR 1.24; 95% CI 1.19, 1.29; p<0.001) of LBW. Moreover, women who had any health insurance, either government schemes (aOR 0.87; 95% CI 0.82, 0.92; p<0.001) or privately funded (aOR 0.89; 95% CI 0.83, 0.96; p=0.003) had reduced odds of having a newborn with LBW. An increase in the education level of women, parity, or wealth index also resulted in significantly reduced odds of LBW in the newborn (Table 3).

**Table 3:** Distribution of factors associated with low birth weight (birth weight < 2.5 kg) in newborns

Characteristic	Total	Low birth weight n (%) rowwise	Crude odds ratio (95% confidence interval)	Adjusted odds ratio (95% confidence interval)
Individual				
Age				
<20	10,171	2130 (21.74)	Ref	Ref
20-30	112,510	18,725 (17.25)	0.75 (0.70, 0.81)	0.89 (0.82, 0.97)
31-49	37,428	5511 (15.97)	0.68 (0.63, 0.74)	0.89 (0.81, 0.97)
Highest educational level				
No education	29,214	5579 (19.58)	Ref	Ref
Primary	18,935	3503 (20.21)	1.04 (0.97, 1.11)	1.12 (1.05, 1.20)
Secondary	86,353	13,991 (17.32)	0.86 (0.82, 0.90)	0.96 (0.91, 1.01)
Higher	25,607	3293 (13.23)	0.63 (0.58, 0.66)	0.74 (0.68, 0.81)
Mother's weight			0.99 (0.997, 0.998)	0.99 (0.998, 0.999)
Lifestyle factors				
Mass media exposure frequency				
No exposure	40,705	7762 (19.77)	Ref	Ref
Any exposure	119,404	18,604 (16.49)	0.80 (0.77, 0.83)	0.96 (0.91, 1.01)
Dietary diversity index				
Low	19,243	3473 (18.99)	Ref	Ref
Moderate	107,145	17,843 (17.40)	0.90 (0.85, 0.95)	1.03 (0.97, 1.09)
High	33,721	5050 (16.14)	0.82 (0.77, 0.87)	1.05 (0.97, 1.13)
Tobacco consumption (smoking/smokeless)				
Yes	150,760	24,944 (17.19)	1.23 (1.13, 1.34)	0.91 (0.82, 1.00)
No	9349	1422 (20.34)	Ref	Ref
Household				
Religion				
Hindu	119,550	20,741 (17.53)	Ref	Ref
Muslim	22,420	3501 (16.28)	0.91 (0.86, 0.97)	0.93 (0.86, 0.99)
Other	18,139	2,124 (16.39)	0.92 (0.85, 1.00)	1.06 (0.96, 1.16)
Caste				
Scheduled Caste	31,789	5977 (18.89)	Ref	Ref
Scheduled Tribe	30,519	4387 (17.93)	0.94 (0.88, 1.00)	0.87 (0.82, 0.93)
Other Backward Castes	61,490	10,168 (16.82)	0.87 (0.83, 0.91)	0.95 (0.90, 1.00)
Others	27,170	4355 (16.24)	0.83 (0.78, 0.89)	0.98 (0.91, 1.05)
Health insurance				
None	114,694	19,515 (17.83)	Ref	Ref
Government schemes	30,492	4641 (15.75)	0.86 (0.82, 0.91)	0.87 (0.82, 0.92)
Private funded	14,923	2210 (25.36)	0.84 (0.78, 0.89)	0.89 (0.83, 0.96)
Type of residence				
Urban	35,676	5551 (16.24)	Ref	Ref
Rural	124,433	20,815 (17.72)	1.11 (1.06, 1.17)	1.04 (0.98, 1.10)
Region				
North	31,204	5378 (17.87)	Ref	Ref
Central	39,063	7532 (19.22)	1.09 (1.04, 1.15)	0.92 (0.87, 0.98)
East	28,932	4801 (17.04)	0.94 (0.89, 1.00)	0.71 (0.66, 0.76)
Northeast	23,374	2684 (14.17)	0.76 (0.70, 0.82)	0.59 (0.53, 0.65)
West	15,325	2762 (18.27)	1.03 (0.95, 1.10)	0.90 (0.83, 0.98)
South	22,211	3209 (14.40)	0.77 (0.72, 0.83)	0.80 (0.74, 0.86)
Wealth index				
Poorest	36,928	7110 (20.47)	Ref	Ref
Poor	36,342	6237 (18.92)	0.91 (0.86, 0.95)	0.89 (0.84, 0.95)
Middle	32,381	5034 (16.70)	0.78 (0.74, 0.82)	0.87 (0.81, 0.93)
Richer	29,572	4459 (15.94)	0.74 (0.69,0.78)	0.77 (0.72, 0.84)
Richest	24,886	3526 (13.95)	0.63 (0.59, 0.67)	0.73 (0.68, 0.80)
Pregnancy related				
Pregnancy intended				
Yes	148,738	24,205 (17.06)	0.82 (0.77, 0.88)	0.83 (0.78, 0.90)
No	11,703	2161 (20.02)	Ref	Ref
Parity				
1	56,614	10,022 (18.43)	Ref	Ref
≥2	103,495	16,344 (16.65)	0.88 (0.85, 0.92)	0.79 (0.75, 0.82)
Anemia				
Severe	3484	698 (21.35)	Ref	Ref
Moderate	46,291	7972 (17.70)	0.79 (0.70, 0.89)	0.86 (0.75, 0.98)
Mild	40,301	6690 (17.70)	0.78 (0.69, 0.88)	0.88 (0.77, 1.00)
Nonanemic	64,141	9995 (16.74)	0.74 (0.66, 0.83)	0.86 (0.75, 0.97)
Previous miscarriage/abortion/stillbirth				
Yes	16,068	2885 (18.67)	1.11 (1.05, 1.17)	1.14 (1.08, 1.20)
No	144,041	23,481 (17.12)	Ref	Ref
Place of delivery				
Public hospital	110,585	18,027 (17.36)	Ref	Ref
Private hospital	38,362	6236 (16.38)	0.93 (0.89, 0.98)	1.12 (1.06, 1.18)
At home	10,830	2048 (21.28)	1.29 (1.20, 1.38)	1.20 (1.11, 1.30)
Other	332	55 (19.85)	1.18 (0.80, 1.74)	1.19 (0.77, 1.83)
Sex of child				
Male	86,100	13,220 (15.95)	Ref	Ref
Female	74,009	13,146 (18.85)	1.22 (1.18, 1.27)	1.24 (1.19, 1.29)
Number of antenatal care visits				
≤4	79,549	13,629 (18.0)	Ref	Ref
≥5	70,843	10,830 (16.0)	0.87 (0.83, 0.90)	0.97 (0.93, 1.01)

**Figure 1:**
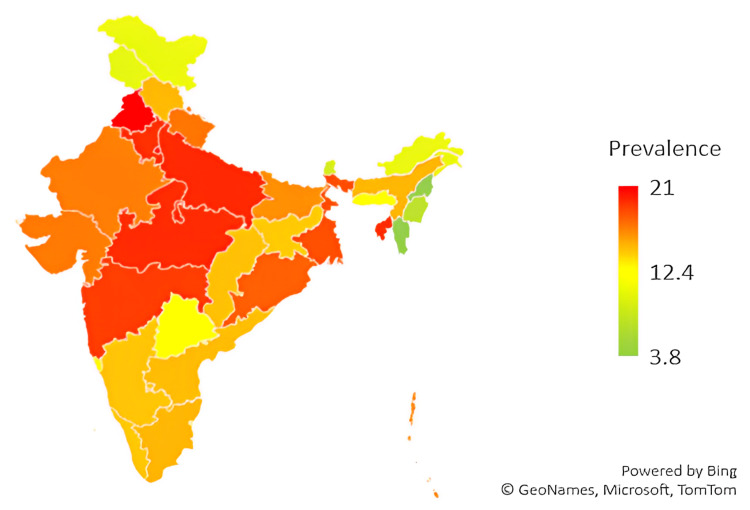
Prevalence of low birth weight in India across states and union territories (NFHS-5, 2019-21) The green color represents regions with a lower prevalence of LBW newborns while the transition to red signifies a higher prevalence of LBW newborns. LBW, low birth weight; NFHS-5, fifth National Family Health Survey.

On regional stratification, a high prevalence of LBW newborns was observed in North and Central India while lower rates were observed in parts of northeastern India (Figure 1).

## Discussion

LBW is an indicator of a multifaceted public health problem [12]. Our study findings indicate that the prevalence of newborns with LBW had a very small decline from 17.5% (17.19, 17.80) in NFHS-4 (2015-16) to 17.29% (17.01, 17.57) in NFHS-5 (2019-21), which is also nearly identical to a previous estimate from a meta-analysis (18%) of studies from India [9,19]. However, the prevalence of VLBW in newborns increased from 1.25% (1.16, 1.33) in NFHS-4 (2015-16) to 2.28% (2.18, 2.38) in NFHS-5 (2019-21). These findings suggest that mothers of newborns with adverse social determinants continue to be at high risk of having LBW and VLBW. 

In the present study, women undergoing pregnancy in their teenage years, those having lower education, and belonging to lower socioeconomic backgrounds had higher odds of having newborns with LBW. These findings are consistent with the results of population-based studies conducted in other LMICs like Ethiopia [20] and Bangladesh [21]. This study also corroborates previous evidence that mothers who smoked tobacco compared to non-smokers had higher odds of having newborns with LBW [22,23].

Regional differences influenced the prevalence of LBW at birth. The country's northeastern, eastern, and southern parts had better birth-weight outcomes compared to other regions, which is consistent with the evidence from the fourth round of the NFHS in India [24]. Higher utilization and quality of ANC services, higher maternal literacy rates correlating with improved awareness, lower parity, and the varied sociocultural phenomenon could be attributed to this phenomenon [25]. Furthermore, in this study, having no health insurance increased the likelihood of having an LBW newborn, a finding akin to that in a study in Mexico although most health insurance in India does not cover outpatient expenses while regardless of insurance status, all government facilities are obliged to provide universal free-of-cost antenatal services to all beneficiaries [26].

In this study, primigravida women had higher odds of having newborns with LBW, a finding also observed in the previous NFHS-4 round, suggestive of possible limitations in dietary adherence and awareness in this vulnerable pregnant group of women [27]. Moreover, this study found that the place of delivery was also associated with LBW in the newborn as those delivering in a public institution had significantly lower odds of LBW compared to those having home delivery or even in private facilities. Similar results were observed in Bangladesh where women who delivered at home were twice as likely to have an LBW baby compared to those delivering in a public facility [28].

Diversity in the diet was also associated with LBW with higher diversity in the diet reducing the odds of having an LBW infant. These findings are in line with a previous study in Ghana [29]. Consumption of diverse and nutritious food has been linked to improved birth outcomes since it is essential for the growth and development of the fetus during a healthy pregnancy while dietary deficiency reduces the birth weight of the newborn. Moreover, a recent study found that stunted mothers had a higher likelihood to have LBW babies [24]. The present study also indicates a higher likelihood of LBW among anemic mothers, which is in accordance with the previous studies indicating the linkage of poor nutritional status of mothers with LBW in their newborns [27]. However, in this study, dietary diversity was ascertained at the time of the survey while LBW outcome is affected by dietary and nutritional status during the period of pregnancy. Therefore, further research is warranted to explore the dietary habits of pregnant mothers in India through prospective studies during pregnancy to understand the effect of reduced dietary diversity on the risk of LBW and other adverse birth outcomes.

Unintended pregnancies increased the odds of having an LBW baby in this study, a finding consistent with the evidence from a systematic review that pooled evidence from studies in Southeastern Ethiopia which found significantly higher odds of LBW among unintended pregnancies resulting in a live birth [30]. Previous evidence also suggests that women who had a previous abortion, miscarriage, or stillbirth were more likely to have newborns with LBW, possibly due to poor care and support [27]. Finally, female newborns had 1.25 times higher odds of having LBW compared to newborns of the male gender suggestive of the phenomenon of a small, although possibly gendered inequality contributing to differential growth.

The major strength of this study is the large, nationally representative study sample. However, there also exist certain study limitations. First, a birth-weight estimation was based on the mother's recall which is subject to bias and was not validated with the written record in nearly 39% of cases which increases the likelihood of underestimation of LBW burden due to rounding off errors in the birth weight. Second, important high-burden risk factors of LBW such as preterm birth, water, sanitation, and hygiene conditions [29], and the extent of social support provided to the mother [26] were not adequately captured in this study. Furthermore, information on the administration of prophylactic antimalarial medicines (IPTp) and the frequency and timing of the medical tests conducted were not available in the dataset. Additionally, as most of the data points are patient-reported, study findings may be subject to a significant recall and social desirability bias, especially those relating to behavioral risk factors. Finally, as this was a cross-sectional survey, causal associations could not be established, and temporality was not derived in this study.

This study has certain important implications and associated recommendations. First, an accurate estimation of LBW in large-scale surveys in India and possibly other LMICs is precluded by the lack of availability of birth-weight records with recent mothers. Consequently, maintenance of digital records with Health Management Information Systems and linking them with the Mother and Child Tracking System should be considered. Second, it is imperative to strengthen the existing maternal and newborn health program components toward achieving improvement in the quality of ANC services for early detection of high-risk pregnancies and reduction of the burden of LBW. Substantial governmental efforts in strengthening nutritional supplementation services and food security initiatives require more focused implementation in antenatal women, especially those from lower socioeconomically disadvantaged backgrounds. Third, growth monitoring and dietary interventions to protect the health of newborns with LBW warrant high prioritization in LMIC settings. 

## Conclusions

Nearly one in six women in India give birth to newborns having LBW with the problem more prevalent in northern and western parts of the country. Adverse social determinants of health including reduced literacy and lower socioeconomic status were independently associated with LBW. Despite increasing investment in increasing nutritional supplementation and food security, the challenge of LBW has mostly remained unchanged in recent years suggestive of the need for prioritizing a multipronged, focused, and evidence-based dietary and nutritional support program for antenatal women to address this major public health challenge. Evaluation and continual monitoring of reproductive and maternal health programs would enable the identification of the current lacunae. Developing integrated strategies within existing public health programs to improve communication for improving awareness of pregnant mothers and improving the quality, coverage, and content of existing ANC services also warrant high prioritization.
